# Prevalence and correlates of common mental disorders among dental students in Brazil

**DOI:** 10.1371/journal.pone.0204558

**Published:** 2018-09-27

**Authors:** Karen Mendes Graner, Antonio Bento Alves de Moraes, Albina Rodrigues Torres, Maria Cristina Pereira Lima, Gustavo Sattolo Rolim, Ana Teresa de Abreu Ramos-Cerqueira

**Affiliations:** 1 Department of Public Health, Medical School of Botucatu, Universidade Estadual Paulista “Júlio de Mesquita Filho”, Botucatu, São Paulo, Brazil; 2 Department of Social Dentistry, Dentistry School of Piracicaba, Universidade Estadual de Campinas, Piracicaba, São Paulo, Brazil; 3 Department of Neurology, Psychology and Psychiatry, Medical School of Botucatu, Universidade Estadual Paulista “Júlio de Mesquita Filho”, Botucatu, São Paulo, Brazil; 4 Department of Basic Life Science, Federal University of Juiz de Fora,Governador Valadares, Minas Gerais, Brazil; University of Queensland, AUSTRALIA

## Abstract

This study aimed to estimate prevalence of common mental disorders (CMD) and associated factors among dental students. In this cross-sectional study, 230 students answered a questionnaire and instruments to assess CMD (Self Reporting Questionnaire-20), hazardous alcohol consumption (Alcohol Use Disorder Identification Test), social support (Social Support Scale), perceptions of academic life (Dundee Ready Education Environment Measure), coping (Ways of Coping Inventory) and resilience (Resilience Scale). Bivariate analysis was conducted using the Chi-Square and Mann-Whitney tests. Logistic regression included all explanatory variableswith p<0.20 in the bivariate analysis, besides sex and academic year. The explanatory variables were analyzed in five successive blocks (backward-stepwise), until all variables presented statistical significance in the final model (p<0.05). The prevalence of CMD was 45.2% (95%CI: 38.7–51.6), with no significant differences between sexes. Students with no extracurricular activities, who had negatively self- assessed their health status and their academic performance, were about four times more likely to present CMD, followed by receiving psychological or psychiatric treatment during university (AOR: 2.65; 95%CI: 1.1–6.1) and those with high scores for confrontive coping (AOR: 1.20; 95%CI: 1.0–1.4). Resilience was a protective factor for CMD among dental students (AOR: 0.93; 95%CI: 0.9–1.0). Aspects related to academic performance, health status and confrontive coping strategies were risk factors to students’ mental health. Individuals with high levels of resilience showed lower prevalence of CMD. Further prospective studies could contribute to understanding the role of resilience among this population.

## Introduction

Global health is an area of study in public health aimed at improving and achieving equity in people’s health. Global mental health is an application of global health in the domains of mental health [1,2]. In 2007, The Lancet published several articles based on empirical research showing the importance of mental disorders in different populations [3–6]. Between 1990 and 2010, the prevalence of mental and behavior disorders increased by 41% [7], representing 7.4% of all global health problems and accounted for a quarter of years living with disability [2,8].

Mental disorders have been frequently observed among college students, especially those in medical areas [9–18]. Common mental disorders (CMD) have been the main focus of research among Brazilian college students, with prevalence rates ranging from 20% to 48%, which are higher than those identified in the general population (22–25%) [10,17,19–24]. According to Goldberg and Huxley (1992) [25], CMD are characterized by anxiety, depression and somatic symptoms that cause important and long lasting functional impairment; however, they are not always associated with a psychiatric diagnosis according to standard criteria, such as those described by the International Classification of Diseases of the World Health Organization (ICD-10).

Several factors have been associated with CMD among university students, including demographic (e.g. sex, family arrangement, income, religion) and social characteristics (e.g. social support, difficulties making friends), and aspects of academic life (academic performance, thoughts of abandoning the course) [10,11,15–17,24]. In college, students face important life changes [26,27], some of which are potentially very stressful [14,28,29].

Some studies involving dental students identified symptoms of anxiety, depression, stress, burnout and even suicide risk[13,14,30,31,32,33,34,35]. A multicenter study [30] developed in seven European dental schools found that psychiatric disorders (36%), burnout syndrome (22%), and other stress-related symptoms (34%) were associated with a negative educational environment. Identifying psychological problems and related factors can benefit college students in various ways, preventing its worsening and favoring positive experiences during academic years [36].

Coping strategies and high resilience are protective factors for mental health [37–40]. Coping is a personal cognitive and behavioral effort to manage internal and external demands perceived as taxing or exceeding an individual’s resources and that interfere with the adaptation process [41]. Resilience refers to positive adaptation [38,42–44], where experiencing trauma or stressful events can help an individual face future adversities and psychological distress [40,45]. Undergraduate students with higher resilience seem to present lower levels of stress [40]. Resilience can also function as a moderator between personality traits and anxiety symptoms. Some active coping strategies seem to predict higher levels of resilience among students [46]. However, few studies so far have investigated the association between mental disorders and these two psychological characteristics in undergraduate students, especially among those in dentistry program, using standardized instruments. Previous studies on dental students investigated specific psychological symptoms (stress, anxiety and depression). This study aimed to investigate the prevalence of CMD among dental students and any associations with demographic characteristics, health status, relational aspects, academic life perceptions and psychological characteristics (ways of coping and resilience) in order to identify potential risk and protective factors. We expected to identify a prevalence of CMD between 30 and 40% [10,17,21,24], higher prevalence of CMD among women [10,11,15–17,24] and resilience as a protective factor [37,38,40].

## Materials and methods

### Subjects

This cross-sectional study included 230 dental students, who voluntarily agreed to participate and were present at the institution on the day the research protocol was applied. The target population at Piracicaba Dental School (University of Campinas; FOP-UNICAMP) was 314 four year program students and the response rate was 73.2%. The students did not receive any incentive for participation.

The research protocol (available on request) included six instruments standardized and validated for use in Brazil and a standardized questionnaire to obtain data on the sociodemographic characteristics, health status, relational aspects, and academic life perceptions of the students (232 questions).

### Outcome variable

#### Common mental disorder

CMD was assessed by the Self Reporting Questionnaire (SRQ-20) [47]. CMD do not provide a psychiatric diagnosis, but enables the evaluation of general mental health status, such as anxiety, insomnia, and other symptoms of mental distress, called ‘common mental disorder’ [48]. There are 20 yes/no questions related to the month prior to the application of the instrument. In the study by Mari and Williams (1986) [49], 80% specificity and 83% sensitivity were obtained, with the cut-off point of 7/8. In this study, different cut-off points were used for men (5/6) and women (7/8) since a lower positive predictive value was determined for men in comparison to women when the cut-off point 7/8 was used for both sexes (66 and 83%, respectively).

#### Exposure variables

**Sociodemographic Characteristics, Health Status, Relational Aspects and Perceptions of Academic Life:** To identify sociodemographic characteristics (sex, age, course year, marital status, living arrangement, family visits, religious practice, scholarship, parental education level, family income, personal allowance), health status (self-assessment of health, practice of physical activity, smoking habit, psychological/psychiatric treatment before entering and during university), relational aspects (difficulty making friends, feelings of rejection, adapted to the city) and academic life perceptions (self-assessment of academic performance, satisfaction with the course, thoughts of abandoning the course, involvement in extracurricular activities) of the students, a standardized questionnaire (36 questions) was applied and the answers were categorized for data analysis.

#### Social support

The Social Support Scale (SSS) [50] was chosen because it showed adequate psychometric properties in a previous study [51]. The scale has 19 items involving five functional dimensions of social support: tangible (provision of practical resources and material help); affective (physical expressions of love and affection); emotional (positive expressions of affection, comprehension and feelings of confidence); positive social interaction (availability of people to have fun or relax with); and information (availability of people to obtain advice or guidance from) [46]. Since the SSS does not have a previously established cut-off point, two categories were created (“sufficient” and “insufficient”) to define the scores in each of the scale’s domains. Therefore, median and interquartile intervals were used. Scores up to the first quartile were classified as “insufficient” support, and scores above the first quartile were classified as “sufficient” support. The continuous scores were used in multivariate analysis.

#### Perceptions of the educational environment

The Dundee Ready Education Environment Measure (DREEM) [52], is a 50-item self-report questionnaire designed to assess students’ perceptions of the educational environment within health care courses in general and medical schools. The DREEM is a validated and reliable inventory [52, 53] and has been used in many studies on health care education throughout the world [54]. The inventory was translated into Brazilian Portuguese and validated for use with medical students. High internal consistency has been reported independently by Cronbach alpha levels of 0.92 and 0.93, respectively. Items in the form of statements relating to the respondent’s course environment (e.g., “I am encouraged to participate in class”) are rated in a 5-point Likert scale, where 4 = strongly agree and 0 = strongly disagree. Nine items are worded negatively (e.g., “Cheating is a problem in this school”) and are reversed scored by the researcher before tallying. Item scores count towards an overall environment score, as well as one of five subscales or domains (abbreviations and maximum subscale scores are in parenthesis): students’ perceptions of learning (SPL, 52), students’ perceptions of teaching (SPT, 44), students’ academic self-perception (SAP, 36), students’ perceptions of atmosphere (SPA, 48), and students’ social self-perception (SSP, 28). The maximum overall DREEM score is 200 and there is not a standardized approach for the analysis of the DREEM scores [55]. In this study, the median was used as a cut-off threshold to categorize the scores as “negative” or “positive” in the bivariate analysis, whereas the total score was used as a quantitative variable in the multivariate analysis [55].

#### Alcohol use

The Alcohol Use Disorder Identification Test (AUDIT) [56] was validated for the Brazilian population [57]. This instrument aims to obtain reliable information about alcohol abuse in the preceding 12 months, especially in relation to the quantity and frequency of alcohol consumption. There are 10 questions, with scores ranging from 0 to 40. Mendonza-Sassi et al. (2003) [58] showed that the cut-off point can vary according to the context and aims of the study. In this study, we adopted ≥8 points to define cases of alcohol abuse.

#### Resilience

The Resilience Scale (RS)[59],adapted for the Brazilian population[60] aims to assess the level of positive psychosocial adaptation of individuals through 25 questions, with answers varying from 1 (strongly disagree) to 7 (strongly agree). The scores range from 25 to 175, with high values indicating higher levels of resilience [60]. Wagnild and Young (1993) [59] reported a reliability coefficient of 0.91 for the scale. In this study, the continuous scores were used for bivariate and multivariate analyses.

#### Ways of coping

The Ways of Coping Inventory (WCI)[61] was developed to assess how individuals deal with internal and external demands when facing a stressful event. The Brazilian version [62] assesses 66 coping strategies for a specific situation. In this study, the situation chosen was “CMD”. In this scenario, the student had to choose one of the following answers to rate each specific coping strategy: 0—Does not apply or not used; 1—Used somewhat; 2—Used quite a bit; and 3—Used a great deal. The coping strategies were grouped into the following scales: Confrontive Coping; Distancing; Self-Controlling; Seeking Social Support; Accepting Responsibility; Escape-Avoidance; Planful Problem Solving; and Positive Reappraisal. In this study, the continuous scores were used for both bivariate and multivariate analysis.

## Data collection and ehical aspecs

The application was previously scheduled with the professors and conducted during regular classes in September 2014. The authors were not the instructors of the students and the questionnaires were applied and analyzed by a PhD researcher. All students enrolled in the dental program were invited to participate. The term of informed consent was explained and distributed with the research protocol to the students in the class. The signed term of consent and the protocol were then inserted in separate boxes to ensure the anonymity of the data. We guaranteed emotional support and referral to the university psychology service to those who asked for this professional assistance, after having answered to the questionnaire. The Botucatu Medical School Research Ethics Committee approved the project in April 2014, under protocol no. 29203514.2.0000.5411.

## Data analysis

The data were analyzed using the Stata 12.0 software (STATA CORP, 2012). Initially, descriptive analyses were performed. The prevalence of CMD was analyzed as a categorical variable (“case” and “non-case”). The Chi-square test was used in the bivariate analyses of the categorical variables, while the Mann-Whitney test was used for quantitative variables. The odds ratios and confidence intervals were also calculated.

**Fig 1:**
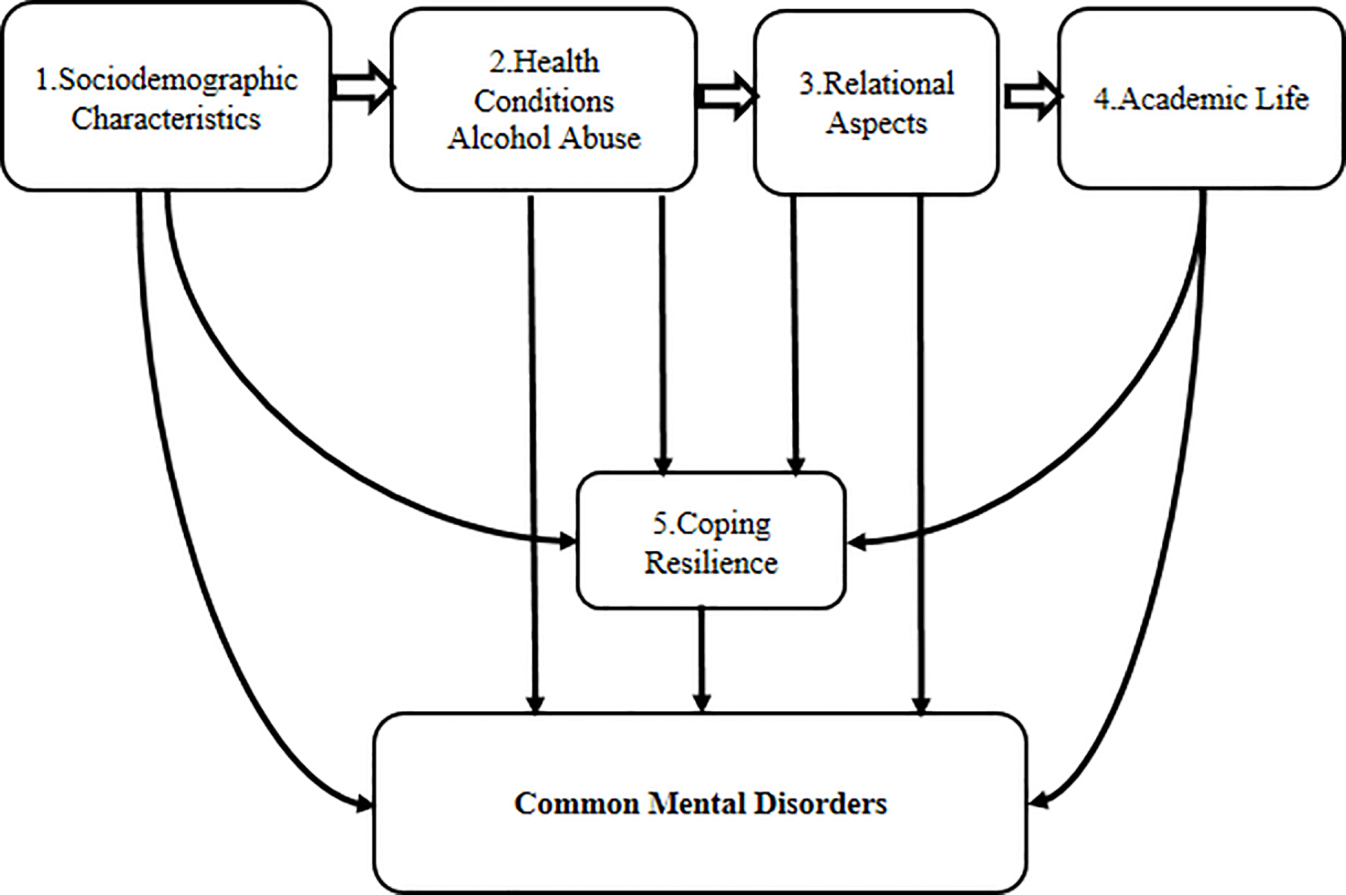
Conceptual framework for common mental disorders.

Next, a logistic regression for successive models was performed, including all variables with a p value <0.20 [63] that did not show multicollinearity, according to the variance inflation factor (VIF value <10). The explanatory variables were distributed in five blocks, considering the conceptual framework for CMD (Fig 1).

Block 1: *sociodemographic variables* (sex, age, marital status, family arrangement, religion, parental educational level, family income, scholarship, personal allowance, working experience in the preceding six months).

Block 2: *health status* (physical activity, self-assessment of health, smoking habit, alcohol abuse—AUDIT, history of psychological/psychiatric therapy before entering and after enrolling in university, and use of psychotropic drugs).

Block 3: *relational aspects* (difficulty making friends, feeling rejected by friends, adapted to the city, frequency of family visits, and social support—SSS).

Block 4: *academic life* (dentistry as first choice, thoughts about quitting the program, self-assessment of academic performance, satisfaction with the academic and the professional choice, under final exam rewrite or retake/repeat, extracurricular activities, history of college hazing, and perceptions of academic life—DREEM).

Block 5: ways of coping (WCI) and resilience (RS).

Based on the diagram above, we hypothesized that the five blocks of explanatory variables would show an influence on the outcome (CMD), and that sociodemographic characteristics (Block 1) could influence the other variables in sequence (health status, relational aspects, perceptions of academic life, coping and resilience). We also hypothesized that health status (Block 2), relational aspects (Block 3) and academic life (Block 4), would influence coping and resilience (Block 5). According to the literature, the last block (Block 5, coping and resilience) included in multivariate analysis was composed of an individual’s characteristics considered as a protective factor for illness development. We hypothesized that these variables could change the results of all antecedent variables.

The models were adjusted for sex and academic year. Given the exploratory nature of the study, in all regression models, the explanatory variables that showed a p value >0.05 were excluded block-by-block (backward-stepwise), until all variables maintained statistical significance (p<0.05). Lastly, the variance inflation factor was calculated for the final model to identify possible multicollinearity.

## Results

Participants included 173 (75.2%) females and 57 (24.8%) males. The mean age was 21 years old (standard deviation ±1.99), ranging from 18 to 34 years old. Regarding marital status, 98.7% were single. Of the total sample, 59.6% lived with friends, 24.8% with parents, and 15.6% lived alone. Most students reported having high family income (≥six minimum wages–MW; 65.9%), monthly expenses up to two MW (87.6%), did not receive a personal allowance or it was insufficient (61.6%), had not worked in the preceding six months (91.3%), and had no scholarship (58.7%). Most participants (70.9%) reported visiting their family every week or every two weeks, having parents (66.1% of fathers and 63.6% of mothers) with a high educational level, and having (84.4%) some a religious practice (data not shown in Table 1).

**Table 1:** Prevalence of common mental disorder according to sociodemographic characteristics, health status, relational aspects and academic life (n = 230).

VARIABLES			CMD		ORCr	95%CI	*P>ǀtǀ*
			n (104)	% 45.2			
SOCIODEMOGRAPHIC CHARACTERISTICS							
	Religion	Yes	84	43,3	1	-	0,17
		No	20	55,6	1,6	0,80–3,36	
	Family income	6 or more MW	60	42	1	-	0,19
		1–5 MW	38	51,4	1,5	0,83–2,57	
	Allowance	Yes	33	37,5	1	-	0,06
		No/Insuficient	71	50,3	1,7	0,98–2,93	
HEALTH STATUS							
	Practice physical activities	Yes	55	38,5	1	-	0,01
		No	47	55,3	2	1,14–3,44	
	Self-assessment of health	Good	52	33,1	1	-	0,00
		Bad	50	70,4	4,8	2,50–9,21	
	Psychological/Psychiatric treatment before entering university	No	49	36,3	1	-	0,00
		Yes	55	57,9	2,4	1,39–4,19	
	Psychological/Psychiatric treatment after entering university	No	30	28,3	1	-	0,00
		Yes	74	59,7	3,8	2,10–6,73	
	Use of psychotropic drugs	No	90	42,5	1	-	0,00
		Yes	14	77,8	4,7	1,47–15,26	
RELATIONAL ASPECTS							
	Difficulty making friends	No	75	38,7	1	-	0,00
		Yes	29	80,6	6,6	2,62–16,50	
	Feelings of rejection	No	74	39,2	1	-	0,00
		Yes	30	73,2	4,2	1,95–9,23	
	Adapted to the city	Yes	26	33,3	1	-	0,01
		No	76	50,7	2,1	1,15–3,66	
ACADEMIC LIFE							
	Thoughts of abandoning the program	No, never	50	35,2	1	-	0,00
		Yes	54	61,4	2,9	1,65–5,17	
	Self-assessment of academic performance	Good	66	37,3	1	-	0,00
		Bad	38	71,7	4,3	2,11–8,60	
	Satisfaction with the course	Satisfied	70	38,5	1	-	0,00
		Not satisfied	34	70,8	3,9	1,90–7,96	
	Extracurricular activity	Yes	19	25,7	1	-	0,00
		No	69	55,2	3,6	1,84–6,89	
	Hazing practices	No	95	43,6	1	-	0,03
		Yes	9	75	3,9	1,0–14,98	

CMD, common mental disorders; Chi-square test, p<0.20; MW, minimum wage; ORCr, crude odds ratio; CI, confidence interval.

The prevalence of CMD was 45.2% (95%CI: 38.7–51.6%), with no significant differences between the sexes (data not shown in Table 1). Table 1 shows the associations between CMD and explanatory variables with *p* values lower than 0.20, which were selected for regression analysis.

Regarding sociodemographic characteristics, none of the associations showed statistical significance (p<0.05) in the bivariate analysis, and the only variables selected to for multivariate analysis, according to the statistical criteria (p<0.20), were *Religion*, *Family income*, and *Allowance*. Regarding health status, the prevalence of CMD was significantly higher for the students who reported no physical activities, self-assessed their health as “bad”, reported psychological/psychiatric treatment before entering and during university, and use of psychotropic drugs. The prevalence of CMD was also higher among participants that reported having difficulties in making friends, feeling rejected by friends, and not adapting or adapting poorly to the city. CMD was also more frequent among those who had thoughts of abandoning the program, self-assessed academic performance as “bad”, were not satisfied with the program, did not participate in extracurricular activities, and applied hazing (Table 1).

The prevalence of CMD was higher among those who reported “insufficient” social support for all domains of SSS (p<0.01) and among students who evaluated the overall educational environment as having more “negative” than “positive” aspects (DREEM; p<0.01) (Table 2). Students with CMD also reported more “negative” than “positive” aspects concerning their perceptions on learning, teachers, atmosphere and social life in university. Only academic perception showed no association with CMD (Table 2). Overall social support (SSS) and perceptions of the overall educational environment (DREEM) were selected for regression analysis (p<0.20).

**Table 2:** Prevalence of common mental disorder according to students’ perceptions of social support (Social Support Scale) and academic life (DREEM) (n = 230).

VARIABLES			CMD		ORCr	95%CI	P>ǀtǀ
			n 104	% 45.2			
SOCIAL SUPPORT SCALE							* *
	Material Support	Sufficient	68	37.2	1	-	*0*.*00*
	Median:15; 4–20	Insufficient	36	76.6	5.5	2.53–12.09	
	Affective Support	Sufficient	73	41.5	1	-	*0*.*04*
	Median: 13; 3–15	Insufficient	31	57.4	1.9	1.02–3.55	
	Emotional Support	Sufficient	73	41.0	1	-	*0*.*02*
	Median: 18; 4–20	Insufficient	31	59.6	2.1	1.12–4.02	
	Informational Support	Sufficient	70	39.5	1	-	*0*.*00*
	Median: 17; 4–20	Insufficient	34	64.2	2.7	1.42–5.25	
	Social Interaction Support	Sufficient	74	40.4	1	-	*0*.*00*
	Median: 15; 3–18	Insufficient	30	63.8	2.6	1.31–5.12	
	Overall Social Support	Sufficient	67	38.7	1	-	*0*.*00*
	Median: 77; 19–90	Insufficient	37	64.9	2.9	1.53–5.56	
DUNDEE READY EDUCATION ENVIRONMENT MEASURE							
	Perceptions of Overall EE	Positive	76	39.6	1	-	*0*.*00*
	Median: 123; 39–195	Negative	28	73.7	4.3	1.91–9.57	
	Perceptions of Learning	Positive	69	39.0	1	-	*0*.*00*
	Median: 30; 6–45	Negative	35	66.0	2.5	1.34–4.68	
	Perceptions of Teachers	Positive	68	39.5	1	-	*0*.*00*
	Median: 27; 4–44	Negative	36	62.1	2.5	1.57–5.90	
	Academic Self-Perceptions	Positive	86	43.2	1	-	0.12
	Median 21; 4–44	Negative	18	58.1	1.8	0.84–3.94	
	Perceptions of Atmosphere	Positive	59	35.3	1	-	*0*.*00*
	Median 29; 6–47	Negative	45	71.4	4.6	2.34–8.93	
	Social Self-Perceptions	Positive	56	33.5	1	-	*0*.*00*
	Median 17; 5–27	Negative	48	76.2	6.3	3.09–13.02	

CMD, common mental disorders; Chi-square test, p<0.20; EE, education environment; ORCr, crude odds ratio; CI, confidence interval.

Regarding the associations between CMD and the psychological characteristics of students, the median of RS scores was lower among students with CMD (non-case: 131 *v* case: 115; p<0.01) compared with those without CMD. Only resilience and confrontive coping variables were included in the logistic regression (p<0.20) (Table 3).

**Table 3:** Median and range scores of Resilience Scale (RS) and Ways of Coping Inventory (WCI) for common mental disorders (SRQ-20).

VARIABLES	Median		P>ǀtǀ
	Case (Min-Max)	Non-Case (Min-Max)	
RESILIENCE	115 (70–161)	131 (94–166)	*0*.*00*
COPING			
Confrontive Coping	6 (0–15)	5 (0–16)	*0*.*05*
Distancing	8.5 (1–18)	8 (0–16)	0.20
Self-Controlling	8 (2–15)	7 (1–15)	0.46
Seeking Social Support	10 (2–18)	9 (2–17)	0.29
Accepting responsibility	12 (13–21)	12 (3–20)	0.64
Escape-Avoidance	4 (0–6)	4 (0–6)	0.83
Planful Problem Solving	7 (1–12)	7 (1–12)	0.49
Positive Reappraisal	14 (4–27)	13 (1–24)	0.78

Mann-Whitney test, p<0.20; Min, minimum score; Max, maximum score.

After bivariate analysis, the blocks of selected variables were included in the logistic regression models, which were also adjusted for sex and academic year, considering 5% significance (Table 4).

**Table 4:** Logistic regression models: Association between common mental disorders and students’ sociodemographic characteristics, health status, relational aspects, academic life and psychological characteristics.

	Explanatory Variables	Model 1		Model 2		Model 3		Model 4		Model 5		Final model	
		OR	95%CI	OR	95%CI	OR	95%CI	OR	95%CI	OR	95%CI	OR	95%CI
**Sociodemographic characteristics**													
	Sex (Female)	1.25	0.65–2.39	0.82	0.40–1.67	0.81	0.38–1.72	1.39	0.56–3.42	0.87	0.35–2.17	0.83	0.33–2.04
	2^nd^ year	1.55	0.71–3.39	0.78	0.32–1.89	0.85	0.34–2.14	0.53	0.17–1.61	0.6	0.19–1.89	0.61	0.19–1.88
	3^rd^ year	1.18	0.71–3.39	0.64	0.26–1.38	0.72	0.30–1.62	0.42	0.15–1.13	0.57	0.19–1.67	0.67	0.23–1.91
	4^th^ year	2.33	0.99–5.46	1.04	0.39–2.78	11.14	0.41–3.19	0.34	0.09–1.26	0.81	0.20–3.31	1.35	0.40–4.59
	Family Income (Low)	1.31	0.69–2.42										
	Allowance (No)	1.38	0.76–2.50										
	Religion (No)	1.61	0.75–3.45										
**Health Status**													
	**Self-assessment of health**			**3.75**	1.92–7.32*	**4.08**	2.07–8.03*	**4.31**	1.95–9.44*	**4.13**	1.80–9.47*	**4.24**	1.87–9.76*
	Physical activity (No)			1.64	0.85–3.15								
	Treatment before entering University (Yes)			1.37	0.71–2.65								
	**Treatment after entering University (Yes)**			**3.41**	0.72–6.86*	**3.26**	1.63–6.54*	**2.89**	1.27–6.59**	**2.5**	1.07–5.84**	**2.65**	1.14–6.13**
	Psychotropic Drugs (Yes)			2.64	0.72–9.72								
**Relational Aspects**													
	Difficulty making friends (Yes)					2.22	0.72–6.86						
	Feeling Rejected (Yes)					2.55	0.95–6.81						
	Adapted to the city (No)					1.38	0.69–2.76						
	Overall Social Support Scale (High)					0.98	0.96–1.01						
**Perceptions of Academic Life**													
	Satisfied with course (No)							1.21	0.46–3.13				
	Thoughts about abandoning the program (Yes)							1.28	0.56–2.92				
	**Self-assessment of academic performance(Bad)**							**3.86**	1.48–9.72*	**3.66**	1.30–10.29**	**3.78**	1.35–10.55**
	Applied hazing (Yes)							4.11	0.69–24.33				
	**Extracurricular Activities (No)**							**3.11**	1.41–6.85*	**4.34**	1.83–10.27*	**4.54**	1.92–10.72*
	Perceptions of Overall Education Environment(DREEM) (High)							0.96	0.94–0.98	0.98	0.95–1.00		
**Psychological characteristics**													
	**Resilience (High)**									**0.94**	0.91–0.97*	**0.93**	0.90–0.96*
	**Confrontive Coping (High)**	** **	** **	** **	** **	** **	** **	** **	** **	**1.19**	**1.05–1.66**	**1.24**	1.05–1.36*

VIF:3.30; CI-Confiance Interval; OR-Adjusted Odds Ratio; DREEM-Dundee Ready Education Environment Measure

*p<0.01

**p<0.05

None of the variables remained associated with CMD in Model 1, (sociodemographic characteristics) and Model 3 (relational aspects) (Table 4). In Model 2 (health status) self-assessment of health and psychological/psychiatric treatment before entering university remained associated with CMD. Self-assessment of academic performance, extracurricular activities and perceptions of overall education environment (DREEM) were associated with CMD in Model 4 (perceptions of academic life). Resilience, confrontive coping, self-assessment of health, psychological/psychiatric treatment before entering university, self-assessment of academic performance and extracurricular activities remained associated with CMD (p<0.05) in Model 5 (coping strategies and resilience). These variables accounted for 37.4% of CMD occurrence (adjusted R^2^ = 0.3749; p<0.01).

Regarding the Final Model (Table 4), no multicollinearity was observed (VIF: 3.30). Extracurricular activities *(No)* (OR: 4.5; 95%CI: 1.9–10.7), self-assessment of health (*Bad*) (OR: 4.24; 95%CI: 1.9–9.8), self-assessment of academic performance (*Bad*) (OR: 3.8; 95%CI: 1.3–10.5), psychological/psychiatric treatment before entering university (*Yes*) (OR: 2.6; 95%CI: 1.1–6.1) and confrontative coping (*High*-WCI) (OR: 1.24; 95%CI: 1.0–1.4) were risk factors for CMD, whereas resilience (*High*) was a protective factor (OR: 0.9; 95%CI: 0.90–0.96).

## Discussion

This cross-sectional study investigated the prevalence and correlates of CMD in Brazilian dental students, considering a combination of sociodemographic characteristics, health status, relational aspects and psychological characteristics.

The SRQ-20 identified that 45.2% of students presented CMD. This prevalence of CMD is higher than among the general Brazilian population [64–67] and lower than that identified in primary care settings [68]. The prevalence identified in this study was similar to that reported in previous national [10,17,23,69] and international studies aimed at undergraduate students [70–72]. Only one study, involving 1,198 university students in Ethiopia, reported a slightly greater prevalence (49.1%) [73], which can be explained by cultural and socioeconomic differences. The high prevalence of CMD identified among students was an important finding considering that psychological distress is one of the leading causes of disabilities [74] and that mental and behavioral disorders represent 7.4% of all health problems [2,8].

The prevalence of psychological distress is usually higher among women [14,24,69,70,75,76]; however, our multivariate analysis showed no significant variation in CMD between the sexes, even though women outnumbered men (75.2%) [31,69,77,78,79]. Among all the variables included in the logistic regression analysis, five characteristics were risk factors for CMD.

Students who made a negative self-assessment of their health showed a higher prevalence of CMD (SRQ-20). In the logistic regression, those who had negative health self-perception showed a four-fold greater risk for CMD. Most students with CMD reported undergoing psychological and/or psychiatric treatment before and during college, a condition similar to that reported by Fiorotti et al. [23]. Our multivariate analysis showed that those students who underwent psychological or/and psychiatric treatment during college showed a three-fold greater risk for CMD. This might be due to certain limitations in the study design, involving a short assessment period (30 days) and its one-way application. Negative academic experiences can lead to psychological distress [9,12,16], a condition that could explain why students seek professional help.

University students are expected to go through changing experiences, such as living alone and far from home, making new friends and adapting to new study approaches. We detected high prevalence of CMD in the students who reported difficulties adapting to the city, relationship problems with peers, little social support, negative perceptions of educational environment, thoughts of quitting the program, and having no extracurricular activities. Such findings are in agreement with those reported in previous studies on psychological distress in university students [10,17,23,70,77,79]. Students with low social support from parents, friends and community are more likely to present psychological distress and experience more difficulties in coping with life adversities and stress [10,17,23,70,79].

A systematic review [14] identified academic aspects as the most prevalent stressors among preclinical and clinical students, influencing their self-evaluation. Negative self-assessment of academic performance was one of the academic characteristics identified as a risk factor for CMD. Stress factors for dental students include concerns about classes, professors and teaching methods [9,16], all of which might interfere with their academic performance and well-being. Psychological distress was found to be higher in students during theoretical and preclinical classes and clinical activities [14]. In this study, although no significant variation was detected for CMD over the four years of the undergraduate program, all multivariate analysis models were adjusted for academic year, given the importance of this variable in the literature. Our multivariate analysis showed that students who reported little satisfaction with extracurricular activities showed a four-fold greater risk for CMD. Involvement in extracurricular activities may result in satisfactory and pleasurable situations and reduce anxiety and stress [80].

This study also aimed to investigate how students tend to cope with academic situations, how resilient they are, and whether such characteristics are associated with psychological distress. Coping strategies are known to mediate emotional responses and changes during stressful situations [81,82] and resilience is defined by positive adaptive responses of individuals facing adversities [83]. According to the literature, some coping strategies, especially resilience, are considered to prevent illnesses [38].

In our multivariate analysis, students who scored for confrontive coping were 1.2 times more likely to have CMD. Confrontive coping style is considered a problem-focused coping strategy [61] and usually includes efforts to manage or change a stressful situation. It can also involve a set of aggressive behaviors; for example: “*I tried to get the person responsible to change his or her mind*; *I expressed anger to the person who caused the problem;* and *I did something that I didn’t think would work”* [61]. This strategy can be due to conflict situations and can favor psychological distress in students. The motivational theory of coping emerged as a motivational and developmental perspective of coping analysis. This theory focuses on self-regulation, considered the ability to control one’s own behavior [84]. Accordingly, confrontive coping is included in a group of opposition behaviors, when individuals view a situation as a threat to their autonomy or their ability to make choices; such conditions can have detrimental health effects [84,85].

Resilience was the only variable identified as a protective factor for CMD [37,38,40]. As expected, students with high scores of resilience showed lower prevalence of CMD, a finding that could be explained by the protective effect of this characteristic on an individual’s health [38]. An individual’s ability to adapt to adversities can favor their well-being [40].

The results showed the SRQ-20 was effective in assessing psychological distress among college students [64]. According to standard criteria, CMD can impair an individual’s life [25]. Identifying risk and protective factors associated with CMD can help reduce distress and encourage positive interactions among university students. The high prevalence of CMD identified in this study is possibly due to a combination of sociocultural aspects, program and students’ personal characteristics. May be offering psychological service and opportunities for extracurricular activities might promote to undergraduate students better perception of their academic performance and wellbeing. However, due to the cross-sectional design, our study did not access the effects of exposure variables on psychological distress over the four-year undergraduate program. Also, we cannot know whether the dentistry program leads vulnerable individuals to psychological distress or attracts individuals with high levels of distress. Moreover, although only four students did not complete the questionnaires in full, the length of the survey, with more than 230 questions, may be another limitation of this study. Longitudinal studies, including other academic, cultural and personal aspects, are required to confirm and expand our findings.
